# Simulating a travel-related origin of *Candida auris* in New York–New Jersey

**DOI:** 10.1128/spectrum.02065-24

**Published:** 2024-12-19

**Authors:** Rita R. Verma, Edward Kiegle, Alexander C. Keyel, Sudha Chaturvedi, Vishnu Chaturvedi

**Affiliations:** 1 Department of Pathology, Microbiology and Immunology, New York Medical College, Valhalla, New York, USA; 2 Wadsworth Center Mycology Laboratory, New York State Department of Health, Albany, New York, USA; 3 Division of Infectious Diseases, Wadsworth Center, New York State Department of Health, Albany, New York, USA; 4 Department of Atmospheric and Environmental Sciences, University at Albany, Albany, New York, USA; 5 Department of Biomedical Sciences, School of Public Health, University at Albany, Albany, New York, USA; 6 Department of Pathology, Westchester Medical Center, Valhalla, New York, USA; University of Iowa, Iowa City, Iowa, USA

**Keywords:** *Candida auris*, pathogenic yeast, outbreaks, antifungal resistance, mobility network

## Abstract

*Candida auris* first appeared in the United States in 2013 in New York–New Jersey (NY-NJ) and led to an unprecedented outbreak since 2016. We hypothesized *C. auris*’ introduction to NY-NJ was not a random event but related to travel patterns between South Asia and NY-NJ. New York City is a US hub for international passengers, including those from South Asia. We tested the hypothesis by simulating introductions to NY-NJ with a Monte Carlo simulation based on travel from South Asia, proportion of US population in NY-NJ, proportion of hospitals in NY-NJ, and finally, proportion of all travelers entering the United States through NY-NJ. The *C. auris* outbreak occurred during increasing travel and trade, and South Asia travel routes predict the distribution of early *C. auris* cases in NY-NJ. The local mobility network within hospitals and extended stay healthcare facilities were also relevant in the spread of *C. auris*. Our observations and simulations link travel patterns to *C. auris* origin and spread and warrant further investigations for understanding the continued spread of the pathogen.

**IMPORTANCE:**

*Candida auris* is an emerging fungal pathogen, with resistance to several antifungal drugs. Serious *C. auris* infections affect hospitalized patients and residents of long-term care facilities, although the pathogen can also be present on a healthy individual’s skin. Many studies have shown international introductions of *C. auris* to the United States. Here, we present a simulation that supports the hypothesis that the earlier introductions of *C. auris* in the New York–New Jersey area are not random but related to travel networks.

## INTRODUCTION

Over the last decade, the yeast pathogen *Candida auris* has rapidly spread in hospitals and healthcare facilities, becoming a major public health concern globally. Through whole genome analysis, various investigators have identified *C. auris* isolates from across the globe, revealing a significant global distribution grouped into four major clades (I–IV) and two minor clades (V and VI). Clade I, or the South Asia clade, covers most isolates from India, Pakistan, and the UK (1, 2); clade II, or the East Asia clade, is from Japan and South Korea (1, 3); clade III is from Southern Africa (e.g., South Africa); and clade IV from South America (Venezuela [1] and Colombia [4]). Researchers have found clade V from Iran (5), and they have reported clade VI from Bangladesh and Singapore (6, 7). Clades I, III, and IV are considered the “outbreak clades” (8). Clade I originated from at least two populations in India and Pakistan. Analysis of cases in India and Pakistan, with the earliest reports dating back to 2009 and 2011 (9, 10) and as early as 2008 in Pakistan, provides crucial insights into the local and global emergence of *C. auris* (1, 2, 11).

**Fig 1:**
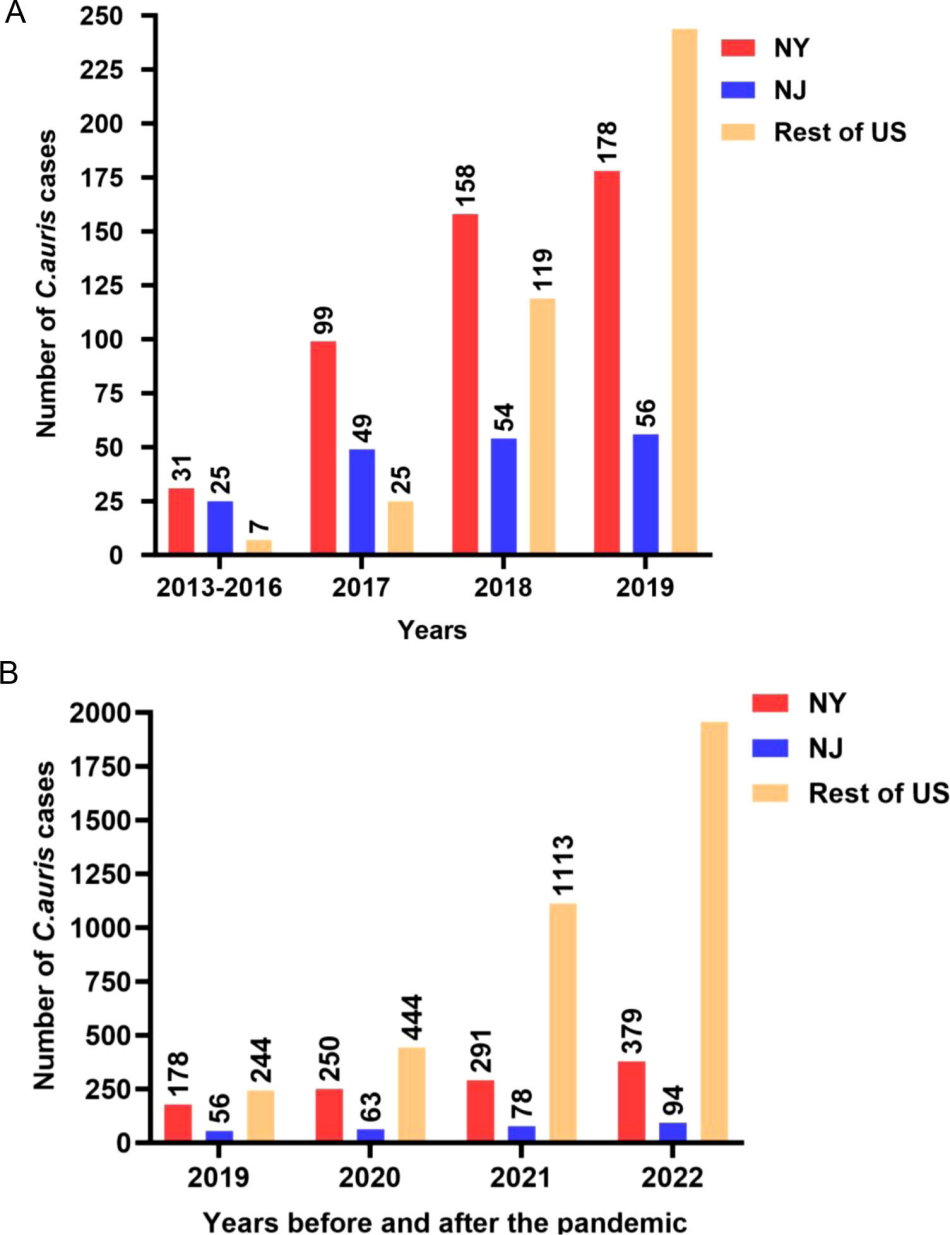
(**A**) *Candida auris* clinical cases in New York–New Jersey and rest of the United States; (**B**) *C. auris* clinical cases after coronavirus disease 2019-related travel restrictions.

**Fig 2:**
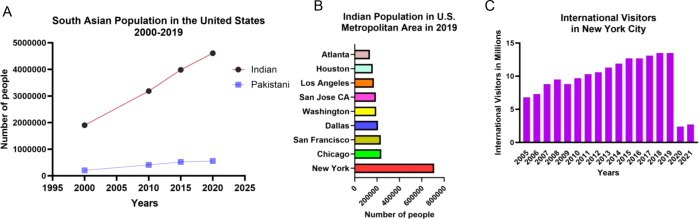
Travel and spread of South Asia clade I *C. auris*. (**A**) Demographic distribution of South Asian ancestry in the US metropolitan areas (2000–2019), (**B**) Indian population in the US metropolitan areas (2019), and (**C**) increase in international visitors in New York City from 2005 to 2021.

In 2016, *C. auris* was first detected in the United States, with retrospective investigation revealing a prior case from 2013. The Centers for Disease Control and Prevention (CDC) issued an alert in 2016 urging laboratories to report *C. auris* isolates to public health authorities (12). Between 2013 and 2016, public health authorities in the New York–New Jersey (NY-NJ) area reported 56 clinical *C. auris* cases, a stark contrast to the 7 clinical cases in the rest of the country (Fig. 1A). The number of *C. auris* cases increased yearly, and as of December 2022, CDC had recorded 5,654 clinical cases of *C. auris* across the country (CDC *Candida auris* tracking, accessed 14 August 2023), with NY-NJ accounting for nearly 20% of cases (Fig. 1B). The comprehensive clinical, surveillance, and genomic analyses showed New York *C. auris* isolates were almost exclusively from clade I, and most were resistant to fluconazole (13, 14). At the time of the updated CDC alert in 2017, when most of the 153 clinical cases in the United States were in the NY-NJ area, the published studies identify at least four likely introductions of clade I C. *auris* into the NY-NJ region between 2013 and 2017. A molecular epidemiology study of 73 clinical cases found that 82% originated from the NY-NJ area and included all but one *C. auris* isolate as clade I (14). The NJ isolates shared a single genetic signature. New York, in contrast, experienced at least three introductions: the unique 2013 case, a 2016 screening case, and the remaining cases in the study (14). The implication of these multiple introductions of *C. auris* to New York on a global scale is not surprising. Since 2005, the United States has emerged as a leading destination for South Asian migration, leading to growing South Asian communities in major US cities (Fig. 2A and B). New York City is a major hub and the most common destination for travelers from South Asia (Fig. 2B and C).

Here, we set out to answer two related questions: “why NY-NJ?” and “why clade I?” We hypothesized a potential role for increased travel and human migration as contributing factors in the emergence of *C. auris*. We simulated introductions to the United States to test the hypothesis that clade I introduction into NY-NJ was not a random chance event but was because of travel networks.

## MATERIALS AND METHODS

### Demographics and *C. auris* cases

The data on *C. auris* clinical cases were acquired from the CDC (https://www.cdc.gov/candida-auris/tracking-c-auris/index.html) and the New York State Department of Health (https://www.health.ny.gov/diseases/communicable/c_auris/).

Demographic distribution data about the South Asian ancestry in the US metropolitan areas were obtained from the Pew Research Center (https://www.pewresearch.org/short-reads/2021/04/29/key-facts-about-asian-americans/). The data about the visitors to the New York City were from The Tourism Industry in New York City, April 2021, Office of the New York State Comptroller (https://www.osc.ny.gov/reports/osdc/tourism-industry-new-york-city).

### Travel data collection

**Fig 3:**
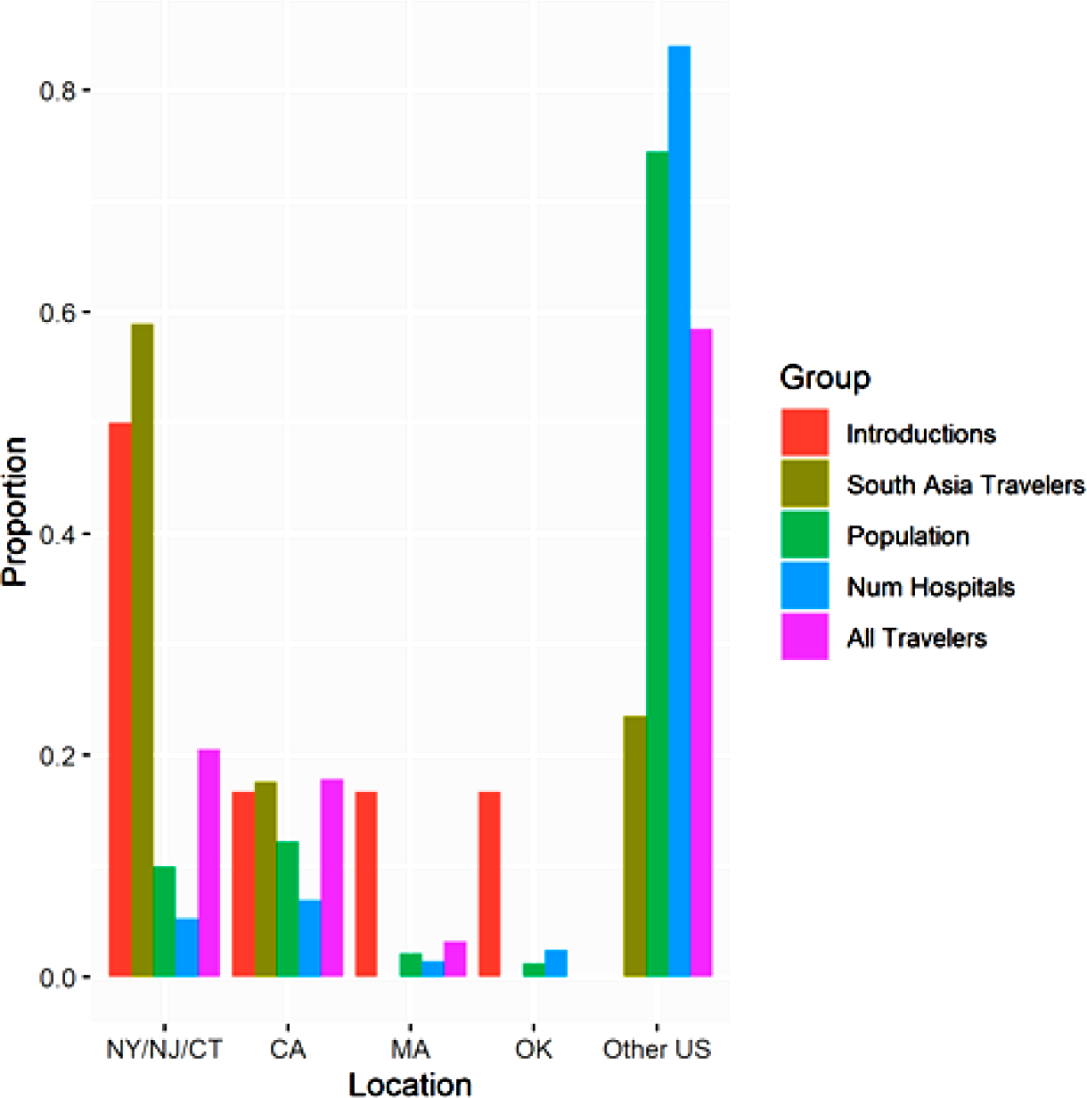
The proportions of clade I clinical *C. auris* introductions, travelers from South Asia, US population, hospitals, and travelers from any foreign country by regions of the United States with *C. auris* introductions, 2015–2017; *C. auris* introductions were most consistent with the number of travelers arriving in the United States from South Asia.

Travel information was obtained from I-92 port of entry forms for New York, New Jersey, Connecticut, Massachusetts, California, Oklahoma, and for the remainder of the United States (15). These locations were chosen based on proximity to clade I clinical cases from 2015 to 2017 reported in reference (14). Travel information was collected from all travelers entering the United States, recording their origin and port of entry. The earliest data available online were from 2019. Data from 2020 and later were not used due to potential disruption to travel patterns due to coronavirus disease 2019 (COVID-19). It was assumed that the proportion of travelers was similar from 2015 to 2017, even if absolute numbers may have differed. Data were pooled for foreign individuals coming to the United States and for returning US citizens and therefore do not imply the national origin or ancestry of the travelers. Mean US population came from the U.S. Census averaged over 2015–2017 by state (16). Hospital data were taken from the American Hospital Association (AHA) survey database (17). The AHA conducts an annual survey of all US hospitals and provides a web summary of number of hospitals (17). While hospital data correspond to 2022, it was assumed that the number of hospitals per state is relatively consistent across years. The analysis was restricted to early introductions of clade I of *C. auris* (2015–2017) documented by reference (14) to reduce the role of domestic transmission following its introductions to the United States. Cases were limited to clinical cases to avoid unequal search effort in screening, leading to the exclusion of a 2016 New York screening case. A 2013 introduction to New York was excluded from the simulation due to a known origin from the United Arab Emirates. A case from Maryland was excluded due to a known domestic travel origin, leading to six introductions in the simulation (three NY-NJ/Connecticut, one Oklahoma, one California, one Massachussets, and zero from the rest of the United States). Cases were pooled for New York, New Jersey, and Connecticut due to their close geographic proximity. The resulting proportions are presented in Fig. 3.

### Monte Carlo simulations

First, we tested whether the observed case proportions were consistent with any of the four sets of proportions (South Asian travel, population, number of hospitals, and all travel) using a *χ*^2^ goodness-of-fit test, using Monte Carlo simulation to calculate *P* values due to the small sample sizes (18–20). Second, a simulation was performed to see if the introduction process could be better represented by modeling introduction events stochastically (21). The simulation was conducted in R version 4.3.2 (18) and code is provided in Supplement S1. Six introduction events were assigned randomly to locations according to each of four sets of proportions (i.e., number of I-92 forms for travelers within the area of interest, percent of US population living within the area of interest, and proportion of hospitals within the area of interest). Simulation runs were scored using mean absolute error (MAE) and compared to a null model predicting that *C. auris* would be absent everywhere, providing a benchmark for uninformative MAE values.

## RESULTS AND DISCUSSION

As New York and New Jersey are populous states, a Monte Carlo simulation (21) was carried out to assess whether the patterns of *C. auris* cases were more consistent with the proportion of travelers from South Asia (15), the proportion of US population (16), the proportion of hospitals (17), or the proportion of travelers from any foreign country (15). Specifically, it was predicted that early *C. auris* introductions would be consistent with the number of travelers arriving in the United States from South Asia but inconsistent with a random distribution based on population or numbers of hospitals. A focus on early cases was chosen to reduce the role of domestic transmission and to reduce the number of cases where the *C. auris* clade was unknown.

**Fig 4:**
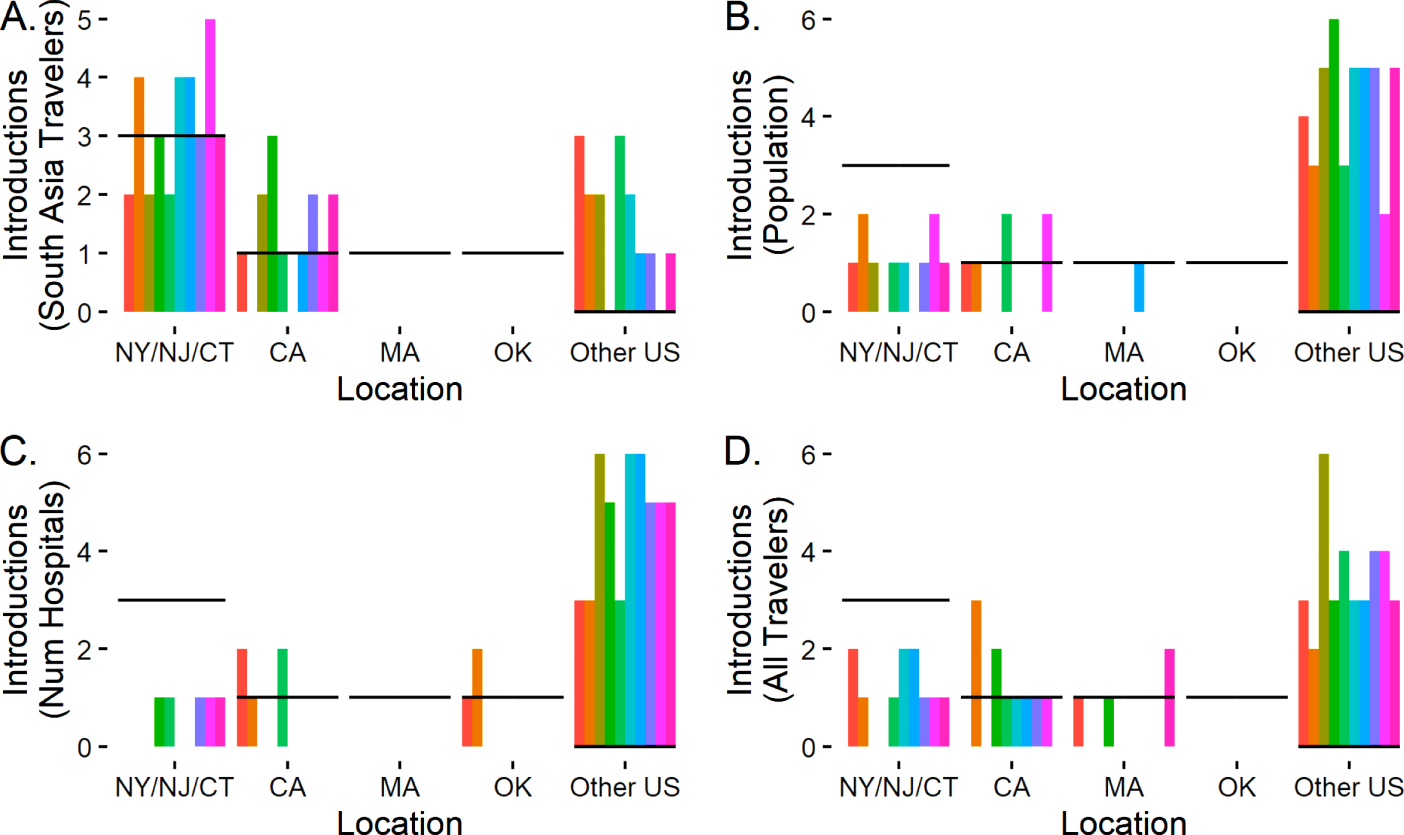
Ten representative examples from Monte Carlo simulations of the introduction of *C. auris* to the United States for (A) travelers from South Asia, (ii) total population, (iii) number of hospitals, and (iv) travelers from any foreign country. The dark bars show the numbers of observed introductions in each region. The simulations demonstrate that population, number of hospitals, and incoming travelers from any foreign countries cannot explain the observed pattern of early *C. auris* cases.

A *χ*^2^ test found that the observed pattern of introductions of *C. auris* clade I significantly differed from all four hypothesized patterns (all *P* < 0.01, Table 1). However, the simulation showed that the proportion of travelers from South Asia produced patterns of cases similar to those observed during the early introduction of *C. auris* to the United States (2015–2017, Fig. 3 and 4). The median MAE values across 100 simulation runs were 4 for travelers from South Asia, 10 for population and number of hospitals, and 8 based on all foreign travelers. Predicting *C. auris* to be absent everywhere would result in a MAE value of 6. While none of the simulations produced a perfect match for the observations, only the simulation based on travelers from South Asia showed improvement over the null model predicting *C. auris* to be absent everywhere (10 examples shown in Fig. 4). In contrast, the simulations based on human population (Fig. 4B), number of hospitals (Fig. 4C), or number of foreign travelers from any country (Fig. 4D) were inconsistent with the pattern of early cases.

**TABLE 1:** Results of four hypothesized patterns of introductions of *C. auris* clade I in NY/NJ (19, 20) (*P* < 0.01)

Scenario	*χ* ^2^	*P* value
Travelers from South Asia	∞	<<0.001
Population	31.8	0.004
Number of hospitals	43.6	0.004
Any foreign travelers	14,010.3	0.0005

We argue that increases in travel have been underappreciated in the independent emergence of *C. auris* from three different continents (1). While there has been a clear increase in agricultural azole use (22) potentially affecting antifungal use in hospital settings (23) and rising global temperatures are decreasing the difference between ambient environmental temperatures and human body temperature (24), there has also been concurrent increases in international travel. For example, international visits to New York City had approximately doubled from 2005 to 2019 (Fig. 2C), providing increased opportunities for fungal pathogens to be imported to the United States. While not evaluated in this simulation, increases in trade could also contribute to the emergence of *C. auris*. Agricultural trade may be particularly relevant, as azole-resistant strains of *C. auris* have been isolated from the surface of apples (25). Retrospective testing of historical fungal samples suggests that the recent emergence of *C. auris* is not just an increase in detection (26, 27).

Prior literature has documented the importance of international travel. For example, the first case of *C. auris* traveling outward from South Asia was to Singapore in 2012, an isolate from a patient who had been transferred from a hospital in India (28). Two more cases, identified as clade I, were transferred to Singapore in 2016–2017 after being hospitalized abroad. From April 2015 to November 2016, a “genetically heterogenous population” of *C. auris* seeded a London hospital, affecting 72 patients (2). What is intriguing is that genomic analysis of patient and environmental samples found that all isolates were from clade I and differed very little from isolates collected in India and Pakistan, though there were no known histories of patient travel (2). This genetic similarity raises interesting questions about the spread and evolution of this pathogen. Within the United States, previous hospital visits in the same Indian city linked individual cases in California and Connecticut, while a single case from Oklahoma involved a patient who had been in a Pakistani hospital (29). Our simulation builds on this evidence to support the idea that the introduction to NY-NJ was not due to random chance but was related to travel networks. Interestingly, the simulation predicts more introductions to the rest of the United States than have been reported to the CDC, suggesting the possibility of unreported or misdiagnosed cases in the rest of the United States prior to 2018.

A limitation of this study is that the country of origin of the flight is not necessarily the country of origin of the traveler, and port of entry is not necessarily the traveler’s final destination. As a consequence, *C. auris* cases were observed in locations (e.g., Oklahoma) that did not correspond to a port of entry but nonetheless were documented to be travel related (14). This made the χ^2^ test an imperfect method of testing this hypothesis, as South Asian travel entries had an expected value of zero case in these locations, leading to division by zero (Table 1). In contrast, the simulation revealed that, while not perfect, using the number of travelers originating in South Asia to predict introductions was more consistent with observations than total population or number of hospitals in an area (Fig. 3).

Sample sizes were too small to repeat the simulation for other clades. We are aware of two introductions of clade II: one to New York (B12043 [14]), which was the port of entry for about 6% of entries to the United States from Japan, and Florida (B14308 [6]), which was the port of entry for <<1% of entries from Japan. The simulation approach would have strongly predicted clade II cases from other locations in the United States. Possible reasons for the discrepancy include within-country travel (port of entry is not final destination) or potentially increased detection effort in these states. Travel from Japan is also more than an order of magnitude greater than travel from South Asia based on the APIS 2019 data set, suggesting that based on travel alone, more clade II introductions would be expected. However, clade II is less virulent (8), so this may account for the decreased number of introductions. Similarly, the introductions of clade III to Indiana (B12631 [14]) and Florida (B18833 [6]) are not consistent with the main port of entry from South Africa, as neither Indianapolis, IN, nor Chicago, IL (which is close to Indiana), are important ports of entry from South Africa (15). The Indiana case was known to be travel related, as the individual was previously hospitalized in South Africa (14). We do not have travel history for the clade III case from Florida, but there are very few travelers entering Florida directly from South Africa. The introduction of clade IV to Florida from a patient with prior hospitalization in Venezuela (14) is consistent with the hypothesis, as prior to the suspension of direct flights between the United States and Venzuela and Florida in 2019 (30), Miami was one of the main ports of entry for individuals coming from Venezuela. The travel suspension prevented running the simulation for clade IV, which also included an introduction from Illinois (B11842 [14]) as the APIS data set only went back to 2019, and therefore, it was not reflective of introductions during the chosen period.

The *C. auris* outbreak in the United States is a complex web of movement of *C. auris* within four sometimes overlapping mobility networks: international transport by air travel, movement between cities, movement between healthcare institutions, and movement within the institutions themselves. Our simulation did not examine domestic or local spread once *C. auris* was introduced to the United States. The ability of *C. auris* to persist on skin and inanimate surfaces and its resistance to common disinfection protocols allow the pathogen to spread extensively within institutions (31). Prior literature has demonstrated the importance of local and domestic spread: of 51 clinical cases in a New York healthcare facility network, 61% had resided in long-term care facilities before admission to the hospital, where *C. auris* was found, and only 8% had traveled internationally in the preceding 5 years. Environmental cultures showed evidence of movement within 15 of 20 facilities tested (12). Transmission between states has also been documented, with a transplanted organ from Illinois resulting in an introduction in Massachusetts, and a *C. auris* case from Maryland resulting from a prior stay at a New Jersey hospital with an active *C. auris* infection (14).

Tracking the movement of *C. auris* within institutions requires the use of genomic tools. A deeper understanding of the “genetic pedigree” of the current collection of New York *C. auris* isolates would give a clearer understanding of how much mobility of *C. auris* can be accounted for at the global, local, or institutional levels. A good example of this is an early study of an outbreak in a London hospital between 2015 and 2016 which affected 72 patients. Their meticulous analysis of the localized outbreak identified multiple genotypes of *C. auris* present on a single patient, implying that several genotypes of *C. auris* had seeded the hospital (2). With the New York outbreak, development of a real-time PCR assay facilitated rapid, large-scale (9,982 samples) patient surveillance in 192 healthcare facilities affected between 2016 and 2018 (13). A recent publication from the CDC, based on extensive genomic analyses, shows *C. auris* spread and transmission rates have worsened since 2021 with more echinocandin-resistant isolates being recognized across the United States (32). To understand the extent of *C. auris* dissemination, a detailed genetic analysis of early New York isolates from 2016 to 2018 is essential. This will illuminate the pathogen’s movement at global, local, and hospital levels.

### Summary and conclusions

In the last 10 years, the spread of multi-drug resistant *C. auris* has become a major public health concern worldwide. Simultaneous and independent emergence of *C. auris* in different parts of the world is the subject of several scientific hypotheses. We hypothesized that travel patterns led to the non-random introduction of clade I isolates in the NY-NJ area. New York City is the US hub for international passengers including those from South Asia, and it has the highest prevalence of *C. auris* clade I. Interestingly, travel resumption post COVID-19 travel restrictions coincided with an increase in *C. auris* spread. Recent resumption of domestic and international travel parallels further *C. auris* spread, although local mobility networks within hospitals and extended stay healthcare facilities remain highly relevant in the spread of *C. auris*. Our observations and simulations link travel patterns to *C. auris* emergence in the NY-NJ area.

## SUPPLEMENTAL MATERIAL

Data S1 spectrum.02065-24-s0001.txt Simulation code.

ASM does not own the copyrights to Supplemental Material that may be linked to, or accessed through, an article. The authors have granted ASM a non-exclusive, world-wide license to publish the Supplemental Material files. Please contact the corresponding author directly for reuse.
